# Lifetime Dual Disorder Screening and Treatment Retention: A Pilot Cohort Study

**DOI:** 10.3390/jcm11133760

**Published:** 2022-06-28

**Authors:** Beatriz Puértolas-Gracia, María Gabriela Barbaglia, Mercè Gotsens, Oleguer Parés-Badell, María Teresa Brugal, Marta Torrens, Lara Treviño, Concepción Rodríguez-Díaz, José María Vázquez-Vázquez, Alicia Pascual, Marcela Coromina-Gimferrer, Míriam Jiménez-Dueñas, Israel Oliva, Erick González, Nicanor Mestre, Montse Bartroli

**Affiliations:** 1 Agència de Salut Pública de Barcelona, 08023 Barcelona, Spain; bpuertolas@imim.es (B.P.-G.); mgotsens@aspb.cat (M.G.); opares@vhebron.net (O.P.-B.); tbrugal@aspb.cat (M.T.B.); ext_ltrevino@aspb.cat (L.T.); ext_crodriguez@aspb.cat (C.R.-D.); ext_jvazquez@aspb.cat (J.M.V.-V.); ext_apascual@aspb.cat (A.P.); ext_mcoromin@aspb.cat (M.C.-G.); miriamjd@gmail.com (M.J.-D.); israelos77@hotmail.com (I.O.); ext_egonzale@aspb.cat (E.G.); ext_nmestre@aspb.cat (N.M.); mbartrol@aspb.cat (M.B.); 2 Institut Hospital del Mar d’Investigacions Mèdiques (IMIM), 08003 Barcelona, Spain; mtorrens@imim.es; 3 Department of Experimental and Health Sciences, Universitat Pompeu Fabra (UPF), 08002 Barcelona, Spain; 4 CIBER Epidemiology and Public Health (CIBERESP), 28029 Madrid, Spain; 5 Biomedical Research Institute Sant Pau, IIB Sant Pau, Sant Antoni Mª Claret 167, 08025 Barcelona, Spain; 6 Red de Investigación en Atención Primaria en Adicciones (RIAPAd), 28029 Madrid, Spain; 7 Faculty of Medicine, Universitat Autònoma de Barcelona (UAB), 08193 Barcelona, Spain; 8 Faculty of Medicine, Universitat de Vic i Catalunya Central, Vic, 08500 Barcelona, Spain

**Keywords:** dual disorder, mental disorders, screening, cocaine use disorder, alcohol use disorder, substance-related disorders, treatment retention

## Abstract

The coexistence of a substance use disorder and another mental disorder in the same individual has been called dual disorder or dual diagnosis. This study aimed to examine the prevalence of lifetime dual disorder in individuals with alcohol or cocaine use disorder and their retention in treatment. We conducted a pilot cohort study of individuals (*n* = 1356) with alcohol or cocaine use disorder admitted to treatment in the public outpatient services of Barcelona (Spain) from January 2015 to August 2017 (followed-up until February 2018). Descriptive statistics, Kaplan–Meier survival curves and a multivariable Cox regression model were estimated. The lifetime prevalence of screening positive for dual disorder was 74%. At 1 year of follow-up, >75% of the cohort remained in treatment. On multivariable analysis, the factors associated with treatment dropout were a positive screening for lifetime dual disorder (HR = 1.26; 95% CI = 1.00–1.60), alcohol use (HR = 1.35; 95% CI = 1.04–1.77), polysubstance use (alcohol or cocaine and cannabis use) (HR = 1.60; 95% CI = 1.03–2.49) and living alone (HR = 1.34; 95% CI = 1.04–1.72). Lifetime dual disorder is a prevalent issue among individuals with alcohol or cocaine use disorders and could influence their dropout from treatment in public outpatient drug dependence care centres, along with alcohol use, polysubstance use and social conditions, such as living alone. We need a large-scale study with prolonged follow-up to confirm these preliminary results.

## 1. Introduction

The coexistence of a substance use disorder (SUD) and another mental disorder in the same individual has been called dual disorder or dual diagnosis (DD) [1]. Several epidemiological studies have shown a high positive association between SUD and other mental health problems [2,3,4]. According to the National Institute for Health and Care Excellence (NICE) [5], the prevalence of DD is estimated to be between 0.05% and 0.2% in the general population. In the clinical population, the prevalence of DD ranges from 34% in mental health care service samples to 46% in drug dependent care service samples. This heterogeneity of DD prevalence estimates could be explained by the distinct health care settings, the primary substance of use, the type of comorbid mental disorder and the assessment method used in DD evaluation [6,7]. Regarding DD evaluation, few validated instruments are currently available to assess DD in people with SUD. The Composite International Diagnostic Interview (CIDI) [8] contains a section to screen for DD; however, the Spanish version of this instrument showed low specificity for the diagnosis of mental disorders in the population of substance users [9,10].

SUDs are most frequently associated with affective, anxiety and personality disorders [11]. For example, individuals with alcohol use disorder (AUD) are three times more likely to develop a depressive disorder in their lifetime than those without this [4]. In addition, between 40% and 73% of people with cocaine use disorder (CUD) would meet the diagnostic criteria for another mental disorder, mainly affective or anxiety disorders [12,13,14]. Individuals with DD have more clinical and social problems than individuals with a single mental disorder. At the clinical level, these individuals show increased psychopathological severity. For example, individuals with dual schizophrenia have more positive symptomatology (i.e., hallucinations, delusions, disorganised speech) [15]. They are also more likely to have infectious diseases (e.g., AIDS, hepatitis or sexually transmitted diseases) [16] and to overdose, with a higher number of hospital emergency department visits and psychiatric hospitalisations than individuals with an SUD alone [15]. In addition, these individuals have an increased risk of premature death, mainly from preventable causes such as suicide [17,18]. At the social level, several studies have suggested that the prevalence of unemployment, homelessness and risk of violent behaviours are higher in individuals with DD [15].

The high complexity of individuals with DD may explain their difficulty in maintaining abstinence or remaining in treatment [19,20,21]. Studies based on health care professionals’ experiences report partial or non-adherence to treatment plans [22,23]. Some studies highlight that individuals with DD are more likely to have more symptoms and medication side effects, polysubstance use, longer substance use, a legal history, less family support, lower socioeconomic status and poor treatment motivation, which have been associated with lower treatment retention. 

However, there are few studies on the topic, and some of these provide contradictory results regarding the prevalence of DD and its influence on treatment retention [15,24,25]. Therefore, according to the previous literature review, our study hypotheses are: the prevalence of lifetime DD in a drug dependence care setting would be around 50%; sociodemographic and clinical characteristics and treatment retention would differ between individuals screening positive for lifetime DD and individuals with a SUD alone; and differences in treatment retention among patients screening positive for lifetime DD and patients with a SUD alone would be explained by some sociodemographic, clinical and follow-up characteristics.

The present study aimed to examine: (i) the prevalence of lifetime DD in individuals with AUD or CUD admitted to treatment in four public outpatient drug dependence care centres in Barcelona (Spain); (ii) the sociodemographic and clinical differences between individuals screening positive for lifetime DD and individuals with AUD or CUD alone; (iii) the differences in treatment retention between individuals screening positive for lifetime DD and individuals with AUD or CUD alone; and (iv) the factors associated with treatment retention during the study period from January 2015 to February 2018.

## 2. Materials and Methods

### 2.1. Design and Study Population

This was a retrospective/prospective dynamic pilot cohort study comprising all inhabitants of Barcelona (Catalonia, Spain) aged ≥18 years admitted to treatment in 4 public outpatient CAS (Catalan acronym for drug dependence care centres) in Barcelona. The study was based on the first years after the implementation of a DD screening interview in the routine clinical practice of these 4 outpatient drug dependence care centres (from a total of 6) managed by the Public Health Agency of Barcelona. We started the study in January 2017, the cohort was identified and assembled at an earlier point in time based on existing Electronic Health Records (EHR), and was followed prospectively until August 2017 (total follow-up time = 38 months). This was a dynamic cohort because patients could be recruited or leave the cohort at different times. These centres offer the following services: biopsychosocial diagnosis; harm reduction; individual, group and family therapy; psychopharmacological treatment; social and occupational assistance; legal advice; health education; and coordination with other social and health care services. The therapeutic programmes of the CAS include alcohol, heroin, cocaine, cannabis, DD and severe addictive disorders. The teams are multidisciplinary with psychiatry, general medicine, psychology, nursing, social work, and social education professionals [26]. 

The study population included individuals meeting AUD or CUD criteria of the International Classification of Diseases Tenth Edition (ICD-10) [27] and screened with the Dual Diagnosis Screening Interview (DDSI-IV) [28]. We excluded individuals who started treatment by court order. We used a non-probabilistic sampling. Individuals admitted to treatment for AUD or CUD were included in the study by convenience, i.e., as a pilot study, the lifetime DD screening was administered according to staff capacity in the centres, and mostly to those individuals who showed or reported psychiatric symptoms. The first admission to treatment during the recruitment period (January 2015–August 2017) was considered as an incident case, regardless of whether the individual had been in treatment before the cohort.

### 2.2. Information Sources

We used the centralised Electronic Health Record (EHR) system of the public Drug Dependence Care Centres of Barcelona, which is managed by the Public Health Agency of Barcelona. Sociodemographic and clinical information of all patients was collected using a standardised survey that is routinely administered during the first treatment visit. We used the Dual Diagnosis Screening Interview (DDSI-IV) [28] to screen for lifetime DD. This brief structured interview of 63 items screens for 11 lifetime mental disorders: depression (7 items), dysthymia (2 items), mania (5 items), panic disorder (3 items), generalised anxiety disorder (3 items), specific phobia (7 items), social phobia (2 items), agoraphobia (2 items), psychosis (24 items), post-traumatic stress disorder (2 items) and attention deficit hyperactivity disorder (6 items), according to the criteria of the Diagnostic and Statistical Manual of Mental Disorders (DSM), 4th version. The DDSI-IV is an adaptation of the screening section of the Composite International Diagnostic Interview (S-CIDI) [8]. It includes some questions to differentiate between primary and substance-induced disorders (e.g., psychosis and attention deficit hyperactivity disorder) and is easy to administer in routine clinical assessments. This screening interview was validated in a Spanish population of substance users from health care settings and research units on drugs of abuse (non-health care settings), showing good psychometric properties, with a sensitivity ranging from 0.80 to 0.93 and a specificity ranging from 0.82 to 0.97 depending on the psychiatric disorder [28,29]. The DDSI-IV was administered by a trained psychologist or psychiatrist during the second or third treatment visit at each centre. Individuals were followed-up annually, and their treatment data recorded (e.g., number of visits, therapeutic programme, services received, status and cause of passive status) in the centralised EHR. We followed the STROBE guidelines for reporting observational studies [30].

### 2.3. Variables

The dependent variable was treatment retention, defined as total days in treatment from the first face-to-face treatment visit to treatment dropout. To our knowledge, there is no standard definition of treatment retention. We considered the definition of treatment dropout of the National Plan of Drugs of the Spanish Government [31], which follows the European Guidelines [32], that define dropout as a lack of face-to-face contact between the individual and the treatment centre for 6 months. Each year was reviewed to determine whether the individual was in treatment or not (passive status) and the cause of passive status: dropout, therapeutic discharge, referral, or exitus (Latin language term indicating the death of the patient). The treatment procedures protocol of the Barcelona Public Health Agency defines therapeutic discharge as occurring when the individual in treatment has a favourable outcome, without compulsion or thoughts about future or occasional drug consumption, at least in the last 6 months before the date of discharge; referral when the individual is referred to another health service; and exitus when the patient dies. Individuals in treatment at the end of the study follow-up were censored at the end date (28 February 2018). The primary explanatory variable was the result of the DDSI-IV. Other covariates were sociodemographic (sex, age, educational level, living arrangements, employment status, and legal history), clinical (substance of use, frequency and years of substance use, previous substance use treatment, previous psychiatric treatment, medical or psychiatric history, family history of substance use, self-perceived health and treatment centre) and follow-up (number of visits with a physician or psychiatrist, psychologist, or social worker during the study period) (Appendix A, Table A1).

### 2.4. Statistical Analysis

We conducted a descriptive analysis of the sample characteristics. We stratified the analyses by the DDSI-IV result, a positive result for one or more mental disorders (dual disorder) or a negative result (AUD or CUD alone, no dual disorder). Sociodemographic and clinical differences between individuals screening positive for DD and individuals with AUD or CUD alone were assessed using Pearson’s chi-square test or Fisher’s exact test for qualitative/categorical variables, and Student’s *t*-test or the Mann–Whitney U test for quantitative variables, using an alpha significance level of 0.05. We estimated Kaplan–Meier survival curves to analyse differences in treatment retention between individuals screening positive for DD and patients with AUD or CUD alone. We studied whether differences were statistically significant using the Wilcoxon and Log-Rank tests.

A multivariable Cox regression model was estimated and was adjusted for potential confounders. Firstly, we estimated a model with the significant variables (*p*-value < 0.2) in the descriptive analysis. We used a manual backward elimination method and theoretical criteria to construct 4 blocks of variables introduced in the model in the following order: explanatory, sociodemographic, clinical and follow-up variables. The final model included explanatory variables (DDSI-IV result and substance of use), sociodemographic variables (sex, age and living arrangements), clinical variables (previous psychiatric treatment) and follow-up variables (visits with a physician/psychiatrist, psychologist or a social worker). Finally, we checked whether the final model met the Cox proportional hazards assumption. We performed all analyses using STATA 14.0 (Lakeway Drive College Station, TX, USA) statistical software.

## 3. Results

### 3.1. Sociodemographic and Clinical Characteristics

The study sample consisted of 1356 individuals with AUD or CUD screened for lifetime DD with the DDSI-IV. This study sample represented 48.0% of the total number of individuals who started treatment due to AUD or CUD in the four CAS during the study period. The prevalence of individuals screening positive for lifetime DD was 74.0% (*n* = 1000). Among these, the lifetime comorbid mental disorders were depression (76.4%), dysthymia (27.2%), mania (13.1%), panic disorder (37.5%), generalised anxiety disorder (26.5%), specific phobia (13.4%), social phobia (17.9%), agoraphobia (13.2%), psychosis (30.1%), post-traumatic stress disorder (23.5%) and attention deficit hyperactivity disorder (19.3%). A total of 71.4% (*n* = 971) were individuals with AUD and 77.5% (*n* = 386) were individuals with CUD (data not shown in Table 1). 

Table 1 details the individuals’ sociodemographic and clinical characteristics. Compared with individuals screening negative for lifetime DD, those screening positive at baseline were more frequently women (30.0% vs. 17.0%, *p*-value < 0.001), younger (56.0% vs. 49.0%, *p*-value = 0.049), unemployed (39.5% vs. 27.3%, *p*-value < 0.001) and reported higher polysubstance use (13.0% vs. 9.0% of alcohol and stimulants, respectively, and 6.0% vs. 3.0% of alcohol/cocaine and cannabis, respectively, *p*-value = 0.006). Moreover, a higher proportion had received previous treatment for an SUD (57.3% vs. 42.1%, *p*-value < 0.001), previous treatment for a psychiatric disorder (45.1% vs. 23.9%, *p*-value < 0.001), and more frequently reported a history of organic and psychiatric problems (35.6% vs. 21.4%, *p*-value < 0.001), a family history of substance use (44.5% vs. 34.3%, *p*-value = 0.002) and poorer self-perceived health (43.6% vs. 28.9%, *p*-value < 0.001). The median number of medical or psychiatric treatment visits (8 [IR: 4–13] vs. 6 [IR: 4–10], social care visits (2 [IR: 1–5] vs. 1.5 [IR: 1–3]) and follow-up time (423 vs. 369 days) were relatively higher and were significant in those screening positive for lifetime DD (data not shown in Table 1). 

### 3.2. Characteristics of Treatment Retention

**Figure 1:**
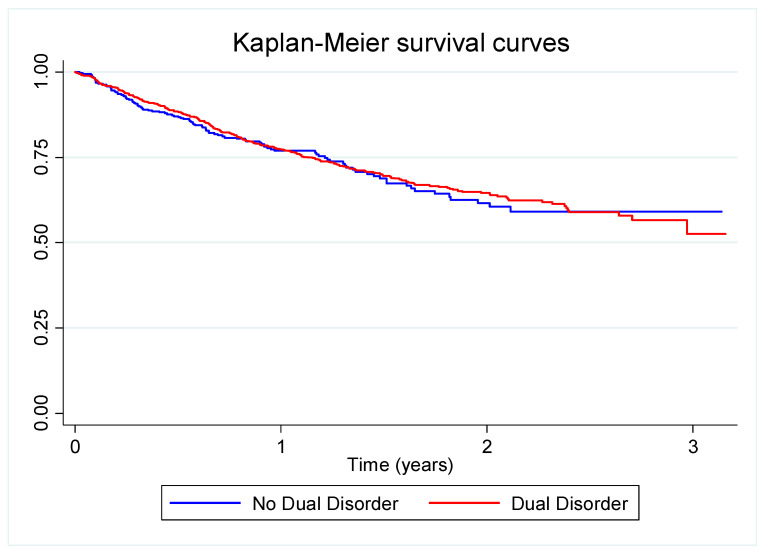
Kaplan–Meier survival curves for treatment retention by DDSI-IV result in a cohort of individuals with alcohol or cocaine use disorder (*n* = 1356). Outpatient drug dependence care centres in Barcelona, January 2015–February 2018.

At one year of follow-up (Figure 1), treatment retention was more than 75% in both groups. Moreover, more than 50% of individuals remained in treatment for the entire follow-up period (38 months). Treatment retention decreased similarly in both groups during the study period, and the difference was not statistically significant (Wilcoxon *p*-value = 0.659; Log-Rank test *p*-value = 0.769). The proportion of dropouts in individuals screening positive for lifetime DD was 29.5% and was 28.4% in those screening negative. There were 458,941 person-days of follow-up among individuals screening positive for lifetime DD and 151,543 person-days of follow-up among those screening negative (Table 1).

### 3.3. Multivariable Explanatory Models of Treatment Dropout

Table 2 shows the different multivariable Cox regression models estimated. After adjustment for different sociodemographic, clinical and follow-up covariates, individuals screening positive for lifetime DD had a 26% increased risk of treatment dropout (HR = 1.26; 95% CI = 1.00–1.60) than those with SUD alone (no DD). According to the substance of use, those who used alcohol only and those who used alcohol or cocaine with cannabis had a 35% (HR= 1.35; 95% CI 1.04–1.77) and a 60% (HR = 1.60; 95% CI = 1.03–2.49) higher risk of treatment dropout, respectively, than those using cocaine only. Individuals who lived alone had a 34% (HR = 1.34; 95% CI = 1.04–1.72) increased risk of treatment dropout than those living with a partner and/or children. The risk of treatment dropout was reduced by 22% with one additional medical visit (HR = 0.78; 95% CI = 0.75–0.80), by 4% with one additional psychologist visit (HR = 0.96; 95% CI = 0.94–0.97) and by 3% with one additional visit with a social worker (HR = 0.97; 95% CI = 0.95–1.00). The Cox proportional hazard assumption (*p*-Value > 0.05) was observed for all variables included in the final model (model 4), except for the variables of previous psychiatric treatment and number of visits with a physician/psychiatrist and with a psychologist (Appendix A, Table A2).

## 4. Discussion

The main findings of this study were: (i) the high prevalence of positive screening for lifetime DD among individuals with AUD or CUD; (ii) the sociodemographic and clinical differences between individuals screening positive for lifetime DD and those with AUD or CUD alone; (iii) the high treatment retention during the study period; and (iv) the risk of treatment dropout was increased by screening positive for lifetime DD, living alone, alcohol use and polysubstance use. 

The prevalence of individuals screening positive for lifetime DD (74%) is consistent with some previous studies conducted in clinical samples but using diagnostic tests. About 62% [33] to 85% [34] of individuals undertaking outpatient substance use treatment were diagnosed with DD using the Psychiatric Research Interview for Substance and Mental Disorders (PRISM). Another study, which administered the Mini-International Neuropsychiatric Interview, found that about two out of three individuals with CUD or AUD had a lifetime mental disorder (73.4% and 76.1%, respectively) [14,35].

In Spain, there is a gatekeeping system at the primary care level and general practitioners can medicate individuals with psychiatric symptoms. This might explain our finding that 23.9% and 8.7% of individuals screening negative for a lifetime mental disorder reported they had previous psychiatric treatment or a previous psychiatric history. This reinforces the importance of incorporating screening tools with good psychometric properties and DSM-IV-based criteria into specialised primary addiction care to allow better identification of psychiatric comorbidities among individuals with SUD [36].

Treatment retention in the cohort was more than 75% at one year of follow-up. This percentage is higher than that reported in another study in Barcelona [37]. Almost 50% of individuals treated in outpatient drug dependence care centres dropped out at one year of follow-up. These individuals had been referred from a hospital emergency department. However, in our study, more than 43% of individuals sought treatment on their own initiative or by family recommendation.

After adjustment for different covariates, screening positive for lifetime DD, alcohol use, polysubstance use and living alone showed the potential to explain treatment retention in our study. The risk of treatment dropout was modestly (26%) higher in individuals with a positive result for lifetime DD than in those with AUD or CUD alone. However, we could not accept or reject our study hypothesis because we did not find a significant association on the bivariate analysis and the association on the multivariate analysis was almost not statistically significant. The previous literature also found contradictory results related to retention in the treatment of individuals with DD. For example, Daigre et al. (2019) reported that DD was not an associated factor for treatment retention [25]. However, in their study, they only selected patients with prolonged treatment stays. In contrast, other studies showed that DD is related to poor treatment adherence in individuals with SUD [19,20,21,23]. Studies conducted in different health care settings (e.g., outpatient clinics, hospitals, therapeutic communities) concluded that the main obstacle to improving health outcomes in these individuals is the difficulty of enhancing their adherence to therapeutic plans. These studies also highlight several related factors, such as symptom severity, medication side effects, years of substance use, polysubstance use or more unfavourable socioeconomic conditions [15,24]. 

In our study, social living conditions, such as living alone, increased the risk (34%) of treatment dropout. Previous studies also reported a higher risk of treatment dropout when individuals had poor social support or family cohesion, or family conflict. Social and family support has been reported to have a buffering effect on stress related to illness and the treatment process and a motivating effect on treatment follow-up [38]. Likewise, several studies have found an association between social support and recovery in individuals with SUD, showing a reduction in substance use, relapses, stress levels and enhanced general well-being [39,40].

We observed that individuals with alcohol use alone presented a higher risk (35%) of treatment dropout than individuals with cocaine use alone. Likewise, a recent study found that patients with cocaine use and a higher education were more likely to complete treatment than patients with alcohol use [25]. A possible explanation could be the legal status of alcohol use and its advertising and availability in the urban environment [41]. Some studies have observed a positive relationship between the concentration of advertising and sale points of alcoholic beverages and risky alcohol consumption and higher associated morbidity and mortality [42,43]. 

In our study, individuals with polysubstance use of alcohol or cocaine and cannabis had a higher risk of treatment dropout (60%) than those with cocaine use alone. Previous studies have shown a relationship between polysubstance use and worse treatment outcomes and premature dropout [44,45]. For example, polysubstance use hampers treatment adherence, i.e., remembering to take prescribed medications, attend treatment appointments, etc. [46]. Likewise, a previous study reported a relationship between polysubstance use and a lower percentage of therapeutic discharges in DD patients [47].

This study has several limitations. First, the participants were recruited from four public drug dependence care centres (CAS) in Barcelona, and therefore the study results cannot be extrapolated to other contexts with a significant private supply of drug dependence care. However, these centres are distributed across the city and account for approximately 55% of all SUD treatment admissions. Therefore, we believe that different patient profiles are represented in our study. Second, we used a lifetime DD screening instrument (DDSI-IV) to determine the presence of comorbid mental disorders. Consequently, we may have overestimated the prevalence of DD. However, this instrument has shown ease of administration in routine evaluations, was validated in a population of substance users and, when compared with the Psychiatric Research Interview for Substance and Mental Disorders (PRISM), as the gold standard, showed high sensitivity and specificity (≥80%) [28]. Third, we screened for lifetime DD, which might hamper the detection of more significant differences in treatment retention. However, because this was a cohort pilot study, recruitment could only be conducted by convenience, and the DDSI-IV was mostly administered to individuals who showed or reported current psychiatric symptoms when starting treatment. However, the present study has allowed us to identify how to improve clinical interview procedures to introduce DD screening systematically as a part of routine clinical practice (i.e., the DD screening is administered by therapists in training supervised by their referent in the centre). Following this preliminary study, the DDSI-IV has been adapted to the DSM-5 criteria, considering current comorbid mental disorders. However, screening for personality disorders has not been introduced in this version either. Fourth, we were unable to differentiate between primary and substance-induced diagnoses for some of the disorders screened. Therefore, an additional routine assessment was recently introduced during the first treatment visits for individuals screening positive in the DDSI-IV.

The main strengths of this study are the cohort study design, with prospective follow-up of participants, the large sample of a clinical population, the inclusion of several public drug dependence care centres and the use of a centralised EHR system with sociodemographic, clinical and follow-up information. Moreover, the study includes many potential confounders of treatment retention, identified through a comprehensive literature review.

## 5. Conclusions

Our study shows that DD is a prevalent issue among individuals with alcohol or cocaine use disorders and could influence their dropout from treatment in public outpatient drug dependence care centres, along with alcohol use, polysubstance use and social conditions, such as living alone. We have designed a new large-scale study, which introduces all the above changes and an extended follow-up to confirm these preliminary results. We believe that introducing DD evaluation in the routine biopsychosocial assessments of individuals with a SUD when starting treatment could help the design of more tailored treatment strategies and improve the prognosis of those individuals. 

## Figures and Tables

**Table 1:** Sociodemographic, clinical and treatment retention characteristics of a cohort of individuals with alcohol or cocaine use disorder (*n* = 1356) by DDSI-IV result. Outpatient drug dependence care centres in Barcelona, January 2015–February 2018.

			Dual Disorder ^1^			No Dual Disorder		
		*n* (%) ^2^	Dropouts ^3^	T ^4^	*n* (%) ^2^	Dropouts ^3^	T ^4^	*p-*Value ^5^
**All participants ^6^**		1000 (74.0%)	295 (29.5%)	458,941	356 (26.0%)	101 (28.4%)	151,543	
**Sociodemographic**								
**Sex**	**Male**	697 (70.0%)	216 (31.0%)	312,230	297 (83.0%)	92 (31.0%)	124,244	<0.001 * ^9^
	**Female**	303 (30.0%)	79 (26.1%)	146,711	59 (17.0%)	9 (15.3%)	27,299	
**Age [$\bar{\times }$** **, SD] ^7^**		44.6 (11.1)				46.5 (12.1)		
**Age**	**18–44 years**	555 (56.0%)	179 (32.3%)	243,111	176 (49.0%)	58 (33.0%)	76,605	0.049 *
	**>45 years**	445 (44.0%)	116 (26.1%)	215,830	180 (51.0%)	43 (23.9%)	74,938	
**Educational level**	**Primary education or lower**	272 (27.2%)	69 (25.4%)	126,968	87 (24.4%)	26 (30.0%)	32,479	0.141
	**Secondary or University education**	715 (71.5%)	218 (30.5%)	327,271	268 (75.3%)	74 (27.6%)	118,901	
	*Missing values*	13 (1.3%)			1 (0.3%)			
**Living arrangements**	**Alone**	189 (18.9%)	63 (33.3%)	83,853	58 (16.3%)	20 (34.5%)	26,768	0.140
	**With others**	702 (70.2%)	200 (28.5%)	325,739	265 (74.4%)	71 (26.8%)	110,836	
	**Homeless or institutionalised**	89 (8.9%)	23 (26.0%)	37,248	25 (7.0%)	6 (24.0%)	9630	
	*Missing values*	20 (2.0%)			8 (2.3%)			
**Employment status**	**Employed**	380 (38.0%)	119 (31.3%)	169,839	184 (51.7%)	56 (30.4%)	76,531	<0.001 *
	**Unemployed**	395 (39.5%)	118 (29.9%)	181,709	97 (27.3%)	26 (26.8%)	44,690	
	**Retired and others**	220 (22.0%)	56 (25.5%)	103,913	75 (21.1%)	19 (25.3%)	30,322	
	*Missing values*	5 (0.5%)						
**Legal history**	**Yes**	262 (26.2%)	78 (29.8%)	123,045	79 (22.2%)	25 (31.7%)	36,456	0.060
	**No**	729 (73%)	211 (28.9%)	332,220	277 (77.8%)	76 (27.4%)	115,087	
	*Missing values*	9 (0.8%)						
**Clinical**								
**Treatment initiation**	**Family or self-initiative**	436 (43.6%)	142 (32.6%)	194,750	170 (47.8%)	52 (30.6%)	73,179	0.288
	**health care or social services recommendation**	560 (56.0%)	153 (27.3%)	261,666	184 (51.7%)	49 (26.6%)	77,574	
	*Missing values*	4 (0.4%)			2 (0.6%)			
**Substance of use**	**Alcohol only**	584 (58.0%)	171 (29.3%)	274,360	245 (69%)	69 (28.2%)	100,997	0.006 *
	**Cocaine only**	229 (23.0%)	70 (30.6%)	99,287	66 (19.0%)	17 (25.8%)	30,921	
	**Alcohol + stimulants**	134 (13.0%)	32 (23.9%)	64,471	33 (9.0%)	9 (27.3%)	15,330	
	**Alcohol or cocaine + cannabis**	53 (6.0%)	22 (41.5%)	20,823	12 (3.0%)	6 (50.0%)	4295	
**Substance use frequency**	**No consumption or <1 day/week**	315 (31.5%)	85 (27.0%)	150,490	98 (27.5%)	28 (28.6%)	43,886	0.528
	**Less than daily (weekly)**	273 (27.3%)	87 (31.9%)	122,708	104 (29.2%)	31 (29.8%)	46,745	
	**Daily**	409 (41.0%)	123 (30.1%)	184,001	153 (43.0%)	42 (27.5%)	60,575	
	*Missing values*	3 (0.2%)			1 (0.3%)			
**Substance use in years [ME (IQR) ^8^**		21 (12–30)			21 (12–34)			0.165
**Previous substance use treatment**	**Yes**	573 (57.3%)	169 (29.5%)	261,005	150 (42.1%)	36 (24.0%)	65,839	<0.001 *
	**No**	420 (42.0%)	124 (29.5%)	194,726	205 (57.6%)	65 (31.7%)	84,906	
	*Missing values*	7 (0.7%)			1 (0.3%)			
**Previous psychiatric treatment**	**Yes**	451 (45.1%)	114 (25.3%)	217,925	85 (23.9%)	23 (27.1%)	38,390	<0.001 *
	**No**	446 (44.6%)	147 (33.0%)	192,902	248 (69.7%)	73 (29.4%)	101,804	
	*Missing values*	103 (10.3%)			23 (6.5%)			
**Medical or psychiatric history**	**None**	210 (21%)	65 (31.0%)	93,830	102 (28.7%)	27 (26.5%)	39,198	<0.001 *
	**Organic disease history**	186 (18.6%)	71 (38.2%)	77,934	131 (36.8%)	42 (32.1%)	56,408	
	**Psychiatric disorder history**	202 (20.2%)	49 (24.3%)	97,531	31 (8.7%)	8 (25.8%)	14,145	
	**Organic and psychiatric history**	356 (35.6%)	93 (26.1%)	169,628	76 (21.4%)	19 (25.0%)	33,554	
	*Missing values*	46 (4.6%)			16 (4.5%)			
**Family history of substance use**	**Yes**	445 (44.5%)	141 (31.7%)	200,047	122 (34.3%)	41 (33.6%)	47,591	0.002 *
	**No**	543 (54.3%)	150 (27.6%)	253,615	231 (64.9%)	60 (26.0%)	102,045	
	*Missing values*	12 (1.2%)			3 (0.8%)			
**Self-perceived health**	**Very good or good**	562 (56.2%)	169 (30.1%)	259,066	252 (70.8%)	76 (30.2%)	103,068	<0.001 *
	**Poor, bad or very bad**	436 (43.6%)	126 (29.0%)	198,814	103 (28.9%)	25 (24.3%)	47,677	
	*Missing values*	2 (0.2%)			1 (0.3%)			
**Treatment centre**	**Centre A**	361 (36.0%)	124 (34.4%)	163,590	74 (21%)	31 (41.9%)	32,198	<0.001 *
	**Centre B**	256 (26.0%)	86 (33.6%)	110,029	87 (24%)	34 (39.1%)	31,506	
	**Centre C**	238 (24.0%)	48 (20.2%)	111,855	132 (37%)	26 (19.7%)	60,639	
	**Centre D**	145 (14.0%)	37 (25.5%)	73,467	63 (18%)	10 (15.9%)	27,200	

^1^ Dual disorder: individual diagnosed with a cocaine or alcohol use disorder with a positive result for one or more mental disorders using the Dual Diagnosis Screening Interview (DDSI-IV); ^2^ *n*, number of cases and %, relative frequency; ^3^ Dropouts: absolute values and relative frequencies (%) of people not attending treatment visits for more than 6 months; ^4^ t: time of follow-up in person-days; ^5^ *p*-value: Pearson’s chi-square test or Fisher’s exact test; Student’s *t*-test or the Mann–Whitney U test to analyse differences between individuals with and without a dual disorder; ^6^ All participants: all study participants with a cocaine or alcohol use disorder screened using the Dual Diagnosis Screening Interview (DDSI-IV); ^7^ $\bar{\times }$, Mean and SD, standard deviation; ^8^ ME, median and IQR, interquartile range; ^9,^ *, indicates that differences between individuals with and without a dual disorder are statistically significant (*p*-Value < 0.05).

**Table 2:** Association of sociodemographic, clinical and follow-up characteristics and treatment dropout in a cohort of individuals with alcohol or cocaine use disorder (*n* = 1356). Outpatient drug dependence care centres in Barcelona, January 2015–February 2018.

		Model ^1^ 1		Model ^1^ 2		Model ^1^ 3		Model ^1^ 4	
		HR ^2^	95% CI ^3^	HR ^2^	95% CI ^3^	HR ^2^	95% CI^3^	HR ^2^	95% CI ^3^
**DDSI-IV ^4^ result**	**No dual disorder**	1		1		1		1	
	**Dual disorder**	0.96	0.77–1.21	1.00	0.79–1.25	1.01	0.79–1.28	1.26	**1.00–1.60**
**Substance of use**	**Cocaine only**	1		1		1		1	
	**Alcohol only**	0.95	0.74–1.22	1.12	0.86–1.46	1.20	0.92–1.57	1.35	**1.04–1.77**
	**Alcohol + stimulants**	0.78	0.53–1.12	0.76	0.52–1.10	0.73	0.50–1.08	0.89	0.61–1.30
	**Alcohol or cocaine + cannabis**	1.57	**1.02–2.42**	1.60	**1.04–2.47**	1.62	**1.04–2.51**	1.60	**1.03–2.49**
**Sociodemographic**									
**Sex**	**Female**			1		1		1	
	**Male**			1.34	**1.05–1.72**	1.27	**1.00–1.64**	1.11	0.86–1.42
**Age**	**>45 years**			1		1		1	
	**18–44 years**			1.40	**1.13–1.75**	1.47	**1.17–1.85**	1.10	0.88–1.39
**Living arrangements**	**With others**			1		1		1	
	**Alone**			1.26	0.98–1.61	1.26	0.98–1.63	1.34	**1.04–1.72**
	**Homeless or institutionalised**			0.99	0.67–1.45	0.94	0.64–1.38	0.86	0.58–1.27
	*Missing values*			1.25	0.71–2.20	0.99	0.55–1.77	1.93	**1.09–3.39**
**Clinical**									
**Medical or psychiatric history**	**None**					1			
	**Organic disease history**					1.34	**1.00–1.78**		
	**Psychiatric disorder history**					0.68	0.42–1.08		
	**Organic and psychiatric history**					0.76	0.50–1.17		
	*Missing values*					1.41	0.75–2.66		
**Previous psychiatric treatment**	**Yes**					1		1	
	**No**					0.83	0.56–1.25	1.03	0.82–1.29
	*Missing values*					0.85	0.49–1.47	0.97	0.67–1.40
**Treatment centre**	**Centre A**					1			
	**Centre B**					1.09	0.84–1.41		
	**Centre C**					0.53	0.40–0.71		
	**Centre D**					0.58	0.41–0.81		
**Follow-Up**									
**Physician/Psychiatrist visits**								0.78	**0.75–0.80**
**Psychologist visits**								0.96	**0.94–0.97**
**Social worker visits**								0.97	**0.95–1.00**

^1^ Model: Cox regression model; ^2^ HR: hazard ratio; ^3^ 95% CI: confidence interval at 95% normal approximation; ^4^ DDSI-IV: Dual Diagnosis Screening Interview (DDSI-IV).

## Data Availability

The data that support the findings of this study are not publicly available but are available from the corresponding author (M.G.B) upon reasonable request.

## Appendix A

**Table A1:** Baseline sociodemographic and clinical information routinely collected by survey in outpatient drug dependence care centres in Barcelona.

Sociodemographic Variables	
**Sex**	Male
	Female
**Age**	
**Residence area**	
**Educational level**	Cannot read or write
	Unfinished primary education
	Completed primary education
	Elementary school or ESO
	Upper secondary school, BUP, COU, intermediate professional training
	University bachelor’s degree of 3 years
	University bachelor’s degree of 4 or 5 years
	Other higher education degrees
**Cohabitation**	Alone
	Alone with children
	With parents
	With a partner
	With a partner and children
	With friends
	Other
**Employment status**	Employee with indefinite contract or self-employed
	Employee with a temporary contract
	Unpaid work for the family
	Unemployed without having worked before
	Unemployed having worked before
	Permanent disability or retired
	Student
	Only housework
	Other
**Legal history**	Yes
	No
**Clinical variables**	
**Treatment initiation**	Self-initiative
	Family or friends’ recommendation
	Drug dependence care referral
	Primary care referral
	Emergency department or hospital referral
	Social services referral
	Legal services referral
	Prison or similar
	Company, service of a company
	Other drug dependence services
	Other
**Primary substance of use**	
**Secondary substance of use**	
**Third substance of use**	
**Fourth substance of use**	
**Substance use frequency**	Every day
	4 or 6 days per week
	2 or 3 days per week
	Once a week
	Less than once a week
	No consumption
**Substance use in years**	
**Previous substance use treatment**	Yes, related to the current primary substance of use
	Yes, related to a different primary substance of use
	Yes, related to the current primary substance of use and for other substances
	No, never
**Previous psychiatric treatment**	Yes
	No
**Medical history**	Yes
	No
**Psychiatric history**	Yes
	No
**Family history of substance use**	Yes
	No
**Self-perceived health**	Excellent
	Good
	Regular
	Bad
**Treatment centre**	

**Table A2:** Assessment of the proportional hazards assumption of the final Cox regression model (model 4).

		Rho	Chi-Square	*p-*Value
**DDSI-IV result**	**No dual disorder**	.	.	.
	**Dual disorder**	−0.04967	1.01	**0.314 ***
**Substance of use**	**Cocaine only**	.	.	.
	**Alcohol only**	−0.07545	2.39	**0.122 ***
	**Alcohol + stimulants**	−0.01408	0.08	**0.7767 ***
	**Alcohol or cocaine + cannabis**	−0.02338	0.23	**0.635 ***
**Sex**	**Female**	.	.	**.**
	**Male**	−0.00478	0.01	**0.924 ***
**Age**	**>45 years**			
	**18–44 years**	0.00698	0.02	**0.887 ***
**Living arrangements**	**With others**	.	.	**.**
	**Alone**	−0.03802	0.60	**0.439 ***
	**Homeless or ** **Institutionalised**	0.03603	0.53	**0.468 ***
	*Missing values*	−0.04295	0.75	**0.388 ***
**Previous psychiatric ** **treatment**	**Yes**	.	.	
	**No**	−0.10596	4.49	0.034
	*Missing values*	−0.03373	0.46	**0.** **498 ***
**Physician/Psychiatrist visits**		0.34088	47.90	<0.001
**Psychologist visits**		0.17575	14.19	<0.001
**Social worker visits**		0.05082	1.57	**0.210 ***

*, indicates that the Cox proportional hazard assumption (*p*-Value > 0.05) was observed.
